# Clinical trials are becoming more complex: a machine learning analysis of data from over 16,000 trials

**DOI:** 10.1038/s41598-024-53211-z

**Published:** 2024-02-12

**Authors:** Nigel Markey, Ben Howitt, Ilyass El-Mansouri, Carel Schwartzenberg, Olga Kotova, Christoph Meier

**Affiliations:** 1 Boston Consulting Group, 80 Charlotte Street, London, W1T 4DF UK; 2 Boston Consulting Group, 75 Avenue de la Grande Armée, 75016 Paris, France

**Keywords:** Business strategy in drug development, Clinical trial design

## Abstract

The past decade has seen substantial innovation in clinical trials, including new trial formats, endpoints, and others. Also there have been regulatory changes, increasing competitive pressures and other external factors which impact clinical trials. In parallel, trial timelines have increased and success rates remain stubbornly low. This has led many observers to question whether clinical trials have become overly complex and if this complexity is always needed. Here we present a large-scale analysis of protocols and other data from over 16,000 trials. Using a machine learning algorithm, we automatically assessed key features of these trials, such as number of endpoints, number of inclusion–exclusion criteria and others. Using a regression analysis we combined these features into a new metric, the *Trial Complexity Score*, which correlates with overall clinical trial duration. Looking at this score across different clinical phases and therapeutic areas we see substantial increases over time, suggesting that clinical trials are indeed becoming more complex. We discuss drivers of increasing trial complexity, necessary or helpful (‘good’) complexity versus unnecessary (‘bad’) complexity, and we explore mechanisms of how sponsors of clinical trials can reduce trial complexity where appropriate.

## Introduction

The past decade has seen many innovations in clinical trials, including new trial formats^1^, novel endpoints^2^, new biomarkers and ways of stratifying patients^3^, a broader set of data sources such as real-world data and digital device data^4^, and many others. Also there are new regulatory guidelines which impact the design of specific studies (for example FDA Project Optimus^5^). Furthermore, many sponsors need to conduct clinical trials across more countries and sites to facilitate competitive differentiation and market access. Sponsors are also under pressure to recruit patients faster and to manage costs. In parallel, trial timelines have increased and success rates remain stubbornly low^6^. This has led many observers to question whether clinical trials have become overly complex.

Here we report a large-scale analysis of protocols and other publicly available data from clinical trials over the past 10 years, corresponding to more than 16,000 studies. Using machine learning algorithms, we automatically extracted information about these trials, such as number of endpoints, number of inclusion–exclusion criteria, and others. We then combined these features into a single metric, the *Trial Complexity Score*. To make this actionable and useful for practitioners, we optimized the score such that it correlates with an important trial parameter, namely overall trial duration. We subsequently analyzed trends in complexity score by clinical trial phase, by therapeutic areas, and by disease, to answer the question whether clinical trials are indeed becoming more complex.

## Results

### Construction of the *Trial Complexity Score*

**Figure 1:**
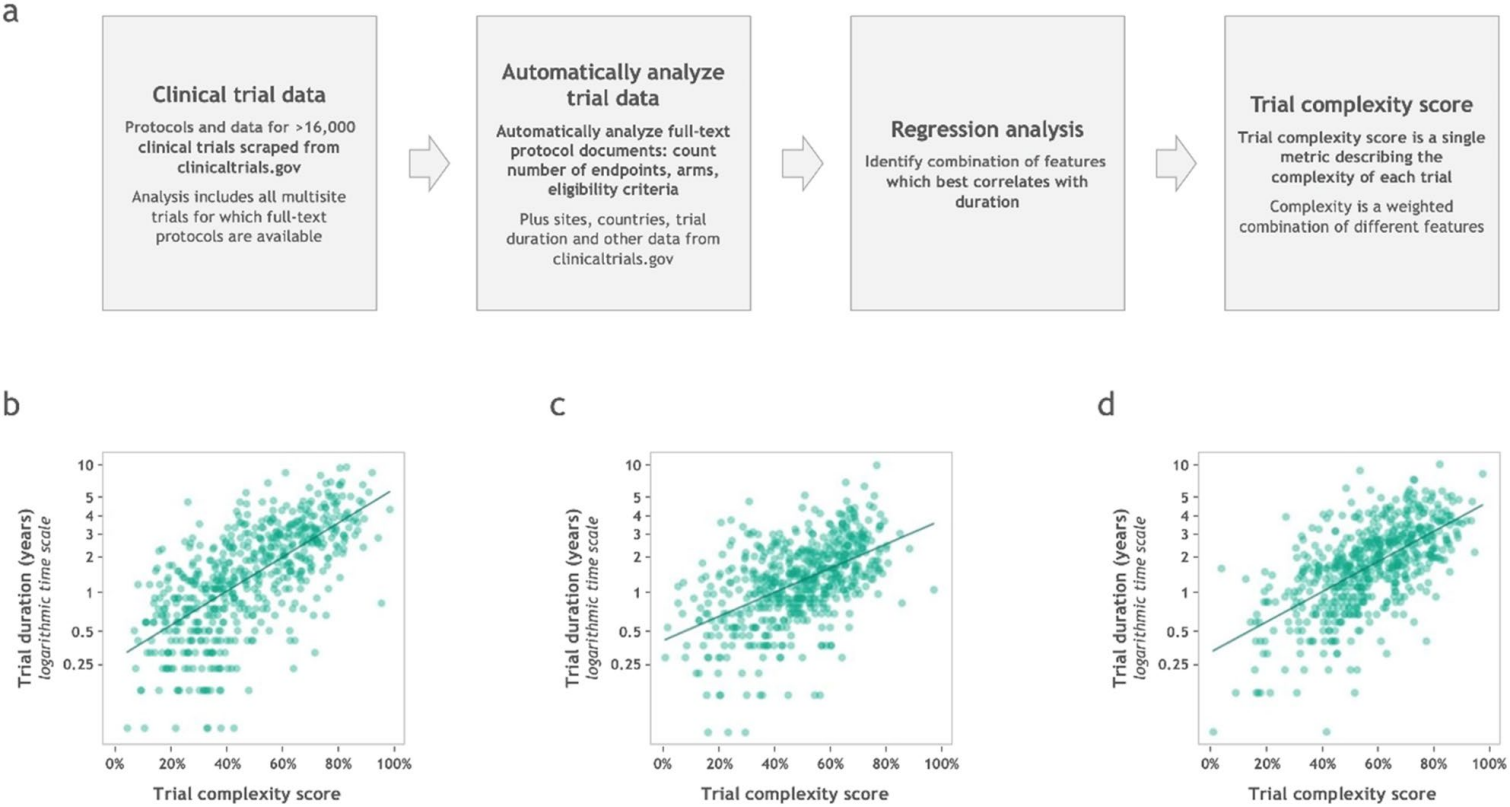
How the Trial Complexity Score is constructed, and how it correlates with trial duration. (**a**) Overview of the process by which clinical trial data was accessed and analyzed, and how the Trial Complexity Scores are calculated. (**b**) Correlation between Trial Complexity Score and phase 1 trial timelines. Slope of the line of best fit (β) is 3.06 which means that a 10 percentage point increase in the score correlates with 36% longer clinical trials overall, compared to otherwise equivalent trials. (**c**) Correlation between Trial Complexity Score and phase 2 trial timelines. β is 2.85 therefore a 10 percentage point increase in the score correlates with 33% longer clinical trials overall, compared to otherwise equivalent trials. (**d**) Correlation between Trial Complexity Score and phase 3 trial timelines. β is 3.01 therefore a 10 percentage point increase in the score correlates with 35% longer clinical trials overall, compared to otherwise equivalent trials.

An overview of how the *Trial Complexity Score* was arrived at is shown in Fig. 1, and a full description is given in the Methods section. Briefly, data (such as number of endpoints, number of inclusion–exclusion criteria, number of study arms, number of sites, etc.) from a large number of multi-site trials were gathered and automatically analyzed. Using a weighted combination of these descriptors, we calculated the *Trial Complexity Score*. The weights were derived using regression, such that the score correlates with overall trial duration—which is of great interest to many sponsors of clinical trials and other practitioners.

Complexity scores range from 0 to 100%, with lower scores corresponding to less complex trials. The score shows a reasonable correlation with clinical trial duration (see Fig. 1). Whilst the details vary slightly by clinical trial phase, this correlation can be summarized in a simple heuristic: A 10 percentage point increase in *Trial Complexity Score* correlates with an increase of overall trial duration of approximately one third.

### Trial complexity is increasing over time

**Figure 2:**
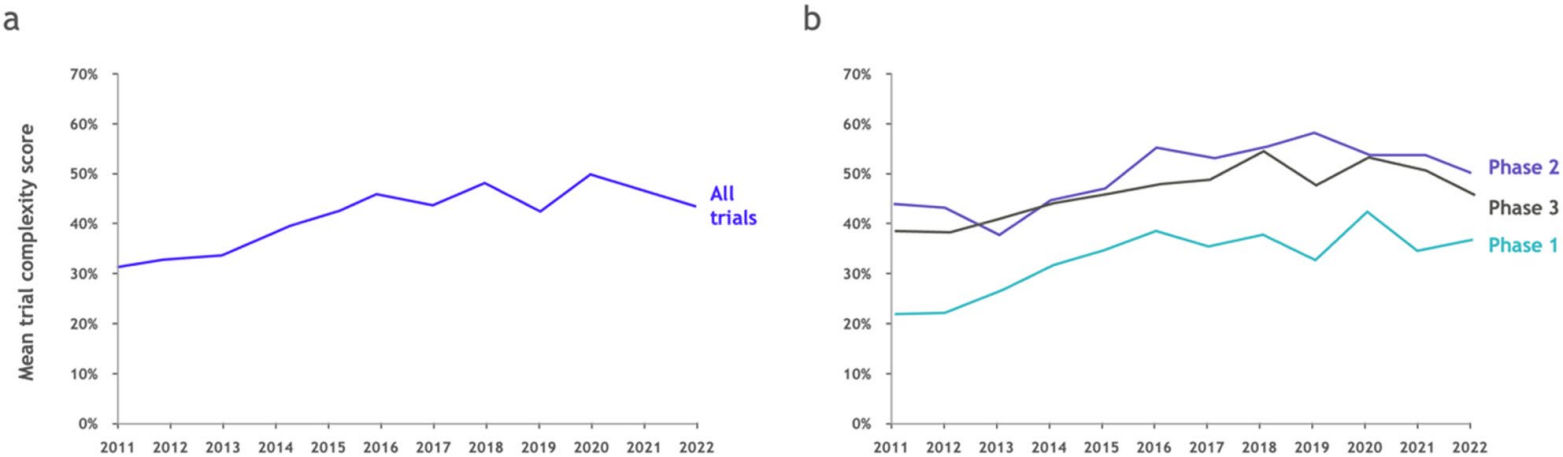
Trial complexity is increasing over time. (**a**) Evolution of mean Trial Complexity Score over time, for all trials. (**b**) Evolution of mean Trial Complexity Score, split by phase.

Looking at *Trial Complexity Scores* across different clinical phases and therapeutic areas, we see substantial increases over time. This suggests that clinical trial complexity, as measured by the score, is indeed increasing (Fig. 2).

Over the last 10 years the average complexity score across all trials has increased by more than 10 percentage points, from the low-30s to the mid-40s. This holds true for all phases, with phase 1 trials increasing from the low-20s to the mid-30s, and phase 2 and phase 3 trials increasing from the mid-40s to the low-to-mid 50s (see Fig. 2).

The increase in phase 1 is particularly pronounced, both in relative and absolute terms. This appears to be driven by increasing complexity of trial designs with more endpoints being assessed, confirming the mantra of some industry observers that “phase 1 is the new phase 3”. It also seems to be driven by pipeline mix, with complex trials e.g. in the oncology space making up a larger proportion of trials.

### Trial complexity by therapy areas (TAs)

**Figure 3:**
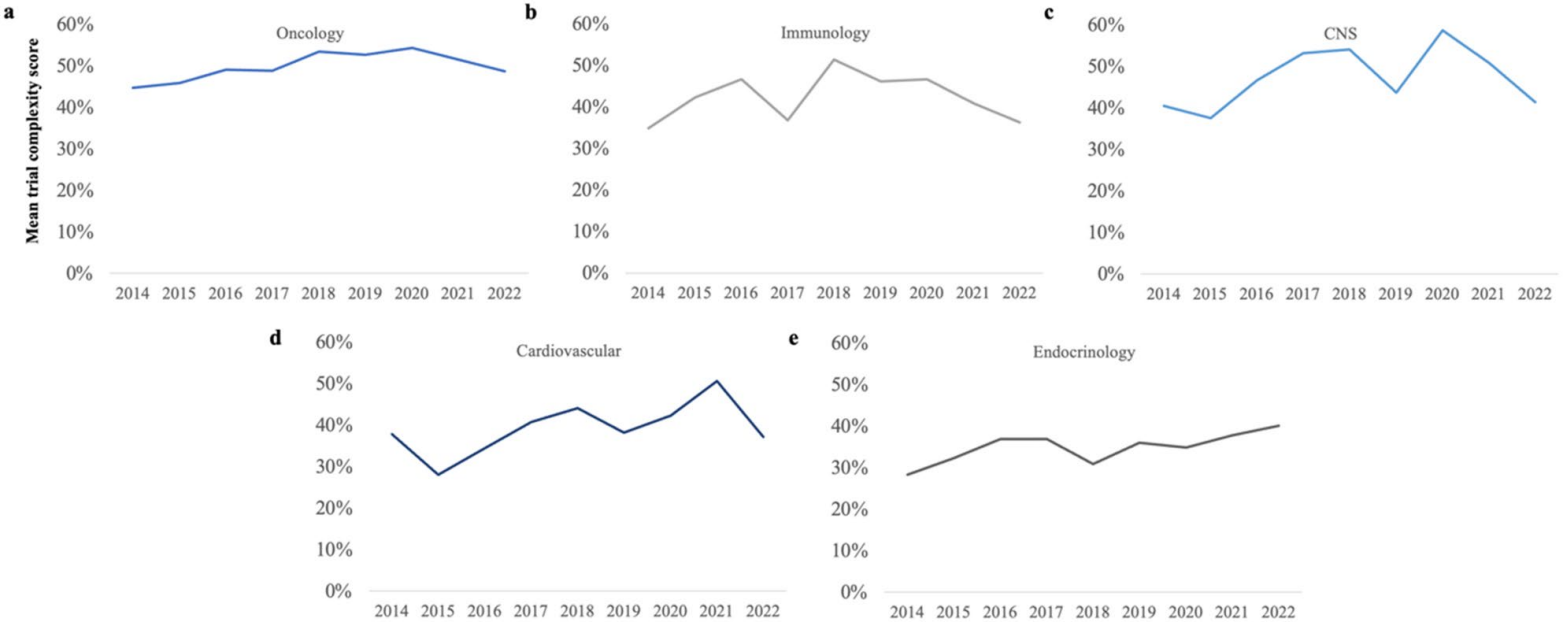
Trends in mean trial complexity, by therapy area (**a**) Oncology; (**b**) Immunology; (**c**) CNS/Neurology; (**d**) Cardiovascular; (**e**) Endocrine and metabolic diseases.

Analyzing *Trial Complexity Scores* by therapy area shows increases across all TAs (Fig. 3).

**Figure 4:**
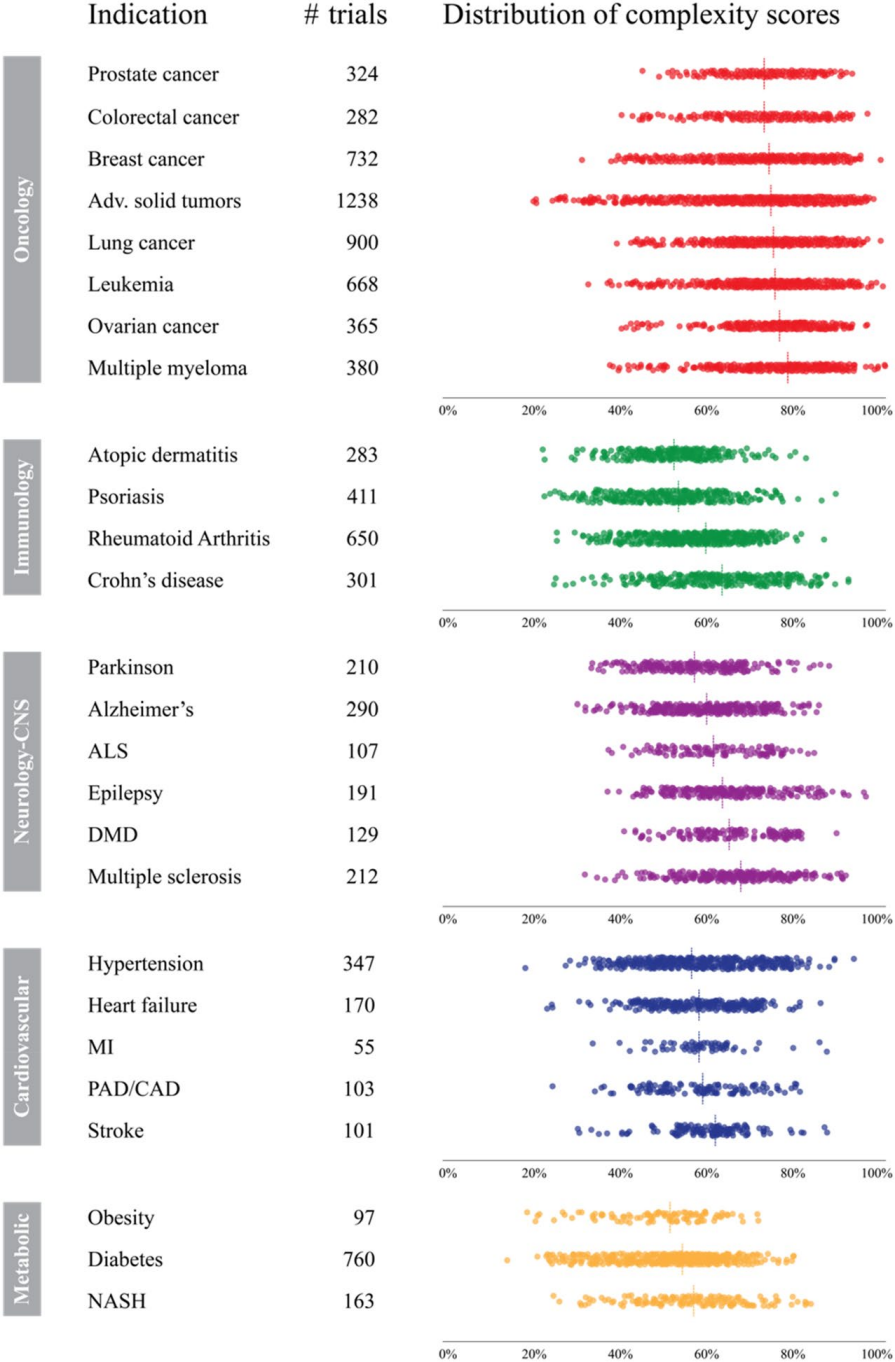
Distribution of Trial Complexity Scores for selected major indications across different therapy areas. Each dot represents one clinical trial. The dashed line indicates the median Trial Complexity Score. Only the most common indications are shown in this view. (*ALS* amyotrophic lateral sclerosis, *DMD* duchenne muscular dystrophy, *MI* myocardial infarction; *PAD/CAD* peripheral artery disease/coronary artery disease, *NASH* non-alcoholic steatohepatitis).

Oncology has historically had the most complex trials, including in major indications such as prostate, colorectal, breast and lung cancer (Fig. 4). Between 2014 and 2020, the average complexity of oncology trials was increasing steadily, but has levelled off since 2020. We believe this may be due to the impact of COVID-19 on trial planning.

Immunology and neurology/CNS trials have historically had average trial complexity, with Crohn’s disease, multiple sclerosis and stroke contributing to more complex trials, and spikes being driven by changes in sponsors and modalities. Cardiovascular trials have seen large increases in complexity up until 2021, driven by a surge in endpoints as wearable devices became widely adopted.

Endocrinology has historically had the lowest complexity, but this has been steadily rising over the last few years, driven by moving from lower complexity indications such as diabetes, to higher complexity indications such as NASH. As with cardiology, there is a rise in digital endpoints as a contributory factor.

## Discussion and conclusion

In and of itself, trial complexity—as defined by our score—is neither good nor bad. There are often legitimate reasons for higher complexity trials, such as the need to address specific unmet medical needs, to ensure rigorous trial design, to address regulatory requirements, or the desire to explore novel scientific objectives. However in some cases complexity may be unnecessary, for example when measuring similar or the same endpoints in different ways; or having an excessive number of exploratory endpoints.

Whatever the reason for trial complexity, it can be associated with increased burdens on patients and investigators, higher costs, and—as our analysis demonstrates—longer timelines to bring medicines to patients. However our analysis also shows that clinical trial complexity is not inevitable, as low-complexity trials exist in every disease area, and for all major indications the spectrum of trial complexities is extremely wide (see Fig. 4). For example, in many major oncology indications, complexity scores range from 20 and 30s to the 90s; and in other therapy areas, scores range from the 20s to the 80s. It is therefore worth examining carefully whether complexity can be reduced in a given trial.

If we apply the heuristic described earlier (namely: a 10 percentage point increase in *Trial Complexity Score* correlates with an increase of overall trial duration of approximately one third), this suggests that trial durations can vary three to fivefold even within the same indication. It also indicates that trial design may reduce trial complexity substantially where this is required, for example in order to accelerate a clinical trial in an indication where there is strong competition.

From our experience, effective strategies to lower trial complexity usually start with systematic evaluation of complexity at the protocol or synopsis review stages. Oftentimes trial design does not take into account the impact on patient and investigator burden, and does not always sharply focus on what is sufficient for approval and competitive market access. Throughout the trial design process, complexity should be assessed regularly to ensure the trial complexity remains under control. In governance forums, trial complexity can be a useful dimension to support decision making about planned trials.

In conclusion our analysis demonstrates that clinical trial complexity (as defined by the *Trial Complexity Score*) has increased across all phases and most therapy areas. This has many consequences, specifically longer timelines to bring medicines to patients, a higher likelihood of protocol changes, higher patient and investigator burdens, increased chances of errors and biases, alongside potential replication challenges. Going forward, sponsors will need to decide if the increases in trial complexity are truly required (e.g. to demonstrate an important outcome or endpoint), whether the consequences of greater complexities are worth it, and how to navigate the cost/speed-versus-complexity trade-off.

### Methods

#### Extraction of clinical trial data

For the trial complexity analysis we extracted and processed clinical trial data, sourced primarily from the ClinicalTrials.gov AACT database, augmented with additional versioned data from the ClinicalTrials.gov web user interface. Most of this data was extracted directly from the AACT database: therapeutic area; number of patients enrolled; number of eligibility criteria; number of endpoints; number of arms. However for some trial-related data we used fuzzy matching algorithms to extract the relevant data: Number of sites; Number of countries.

We did consider including in our analysis other features of clinical trials, such as site characteristics (e.g. geographic distribution of sites; site networks and collaborations between sites; type of sites used), types of data collected (e.g. data from digital devices/wearables; OMICs data), and others (number/frequency of visits, use of an independent Data Monitoring Committee, number of interim analyses, etc.). Whilst these can impact clinical trial complexity, information about these features is often not available in public databases and not systematically recorded. To ensure our analysis is complete and representative, we did not use these features in our analysis.

Our initial dataset encompassed all industry-sponsored interventional trials conducted since 2010. To enhance the reliability and relevance of our analysis, we further refined the dataset by applying several filtering criteria:

- *Initial dataset:* All industry-sponsored interventional trials conducted since 2010 were included. Data for approximately 64,000 trials.
- *Completed trials:* We narrowed down the dataset to trials which are officially listed as completed, reducing the dataset to around 38,000 trials.
- *Minimum trial duration*: We excluded trials with a reported duration of less than one month, leaving approximately 36,000 trials.
- *Outlier removal:* We removed trials with at least one feature greater than five standard deviations from its mean, refining the dataset to around 35,000 trials, and keeping only trials with multiple sites (for which reasonably complete information was available).
- *Top 100 sponsors:* We focused on trials from the top 100 sponsors by the volume of trials, resulting in a final dataset of 16,790 trials.

#### Data processing and feature engineering

Next, we prepared the dataset (above) for model training. This involved grouping our data into two primary types of features: baseline features and design features:

- *Baseline features*, in this context, consist of the therapeutic area and trial phase. These features are categorical in nature and were one-hot encoded, to facilitate their integration into the regression models (below). For trial phase, we split the data set into 3 groups, Phase I, Phase I/II and II, and Phase II/III and III. For Therapeutic area, we mapped the therapeutic area based on the conditions and MeSH terms listed in clinicaltrials.gov. As one-of: Oncology, Immunology, Central Nervous System, Cardiovascular and Metabolic, Respiratory, Muskuloskeletal, and Infectious diseases.
- *Design features* are specific to the protocol and include variables, such as number of endpoints and number of study arms. Each of these features was converted to a percentile ranking i.e. the percentage of trials with features that are less than that score. The model includes the following features: Number of Sites, Patient Enrolment, Number of Countries, Number of Endpoints, Number of Eligibility Criteria, and Number of trial arms. For the target variable we tested different options and found that the logarithm of the trial duration data [extracted from AACT database; computed as the number of months between the trial start date (defined as the date of the original first version of the protocol) and primary completion date] tended to product the best regression results.

Note: In our regression analysis we could have chosen other target variables, such as trial cost or probability of success, but we found the best correlation was with overall trial duration.

#### Regression analysis

Specifically, this analysis we combined a tree-based based model with an Ordinary Least Squares (OLS) model:

- The tree-based model utilized in this study was the Light Gradient Boosting Machine (LGBM) Regressor^7^, which served as the primary model for predicting long-duration based on the set of features discussed in the previous section. This model incorporates non-linear contributions, enabling us to capture complex relationships between the features and the target variable.
- In addition, we employed an Ordinary Least Squares (OLS) Ridge model^8^ to facilitate explainability of each features contribution to the prediction of log-duration. Being a linear model by nature, the OLS Ridge model allowed us to dissect the contribution of each feature’s and further comprehend how non-linearities affect the target variable prediction.

Scatter plots showing the correlation between the *Trial Complexity Score* and duration are shown in Fig. 1. For phase one trials, R^2^ is 44.2% and MAPE (mean absolute percentage error) is 0.637.

For phase two trials, R^2^ is 28.2% and MAPE is 0.387. For phase three trials, R^2^ is 37.2% and MAPE is 0.414.

The full list of features listed in order of relative importance are as follows: therapeutic area; number of sites; number of patients enrolled; number of countries; number of eligibility criteria; number of endpoints; number of arms.

All analyses were carried out in Python^9–13^.

## Data Availability

The data that support the findings of this study are available upon reasonable request to the corresponding author.
